# A study protocol of the photo-supported conversations about the well-being intervention (Be Well™) for people with stress related disorders

**DOI:** 10.1186/s40359-021-00625-3

**Published:** 2021-08-21

**Authors:** A. Birgitta Gunnarsson, Petra Wagman, Hans T. Sternudd, Sara Holmberg, Kristina Holmgren, Ulrica Hörberg

**Affiliations:** 1 Institute of Neuroscience and Physiology, Department of Health and Rehabilitation, The Sahlgrenska Academy, University of Gothenburg, Gothenburg, Sweden; 2 Department of Research and Development, Region Kronoberg, Växjö, Sweden; 3 Department of Rehabilitation, School of Health and Welfare, Jönköping University, Jönköping, Sweden; 4 Department of Music and Art, Faculty of Arts and Humanities, Linnaeus University, Växjö, Sweden; 5 Department of Medicine and Optometry, Faculty of Health and Life Sciences, Linnaeus University, Kalmar, Sweden; 6 Division of Occupational and Environmental Medicine, Department of Laboratory Medicine, Lund University, Lund, Sweden; 7 Department of Health and Caring Sciences, Faculty of Health and Life Sciences, Linnaeus University, Växjö, Sweden

**Keywords:** Activities in everyday life, Health promotion, Instrument, Interviews, Mental health, Photographs

## Abstract

**Background:**

Stress-related illnesses constitute a huge problem in society. The primary care services in Sweden form the first line of care whose role is to coordinate interventions for reducing symptoms, as well as health-promoting interventions. There is lack of knowledge concerning health-promoting interventions for these illnesses. The aim of this study is to evaluate whether photo-supported conversations about well-being (Be Well™) as an intervention, in addition to care as usual within the primary care services, improves health and well-being for patients with stress-related illnesses. The intervention will be compared to a control group, who receive care as usual. A further aim is to conduct a process evaluation.

**Methods/Design:**

This ongoing project has a quasi-experimental design, using quantitative and qualitative methods, and includes patients from primary care centres in two Swedish counties. Seventy patients, 20–67 years, with stress-related illnesses will be recruited. They constitute an intervention group, which receive the intervention together with care as usual, and a control group, which receive care as usual. The intervention, photo-supported conversations about well-being, involves 12 sessions. Care as usual entails medication, occupational therapy, physiotherapy and/or psychotherapy. Data collection is carried out at baseline, and outcomes are assessed directly after the intervention, as well as six months after completion of the intervention. The outcomes are evaluated based on factors related to health, well-being and everyday occupations. Furthermore, data concerning experiences of well-being and perceptions of the intervention will be collected in interviews. The therapists will also be interviewed about their experiences of performing the intervention. Data will be analysed with non-parametric statistics, and qualitative methodology.

**Discussion:**

The project is based on the concept that focusing on well-being despite living with stress-related illness may positively impact health and well-being as well as activity-related aspects, and that photo-supported conversations about well-being can contribute a complement to other treatment and rehabilitation. A strength is the use of a wide range of methods: such as quantitative measures, photographs, and qualitative interviews with participants and therapists. The results will thus provide knowledge about potential effects of this health-promoting intervention.

*Trial registration* Clinical Trials.gov: NCT04832295; retrospectively registered 2nd April 2021 https://clinicaltrials.gov/ct2/show/NCT04832295

## Background

Public health is generally good in Sweden, but despite that it has been reported that levels of well-being are lower [1]. Mental health issues, such as symptoms of depression, anxiety and/or stress, are also increasing internationally [2] and cause large societal costs [3]. Stress-related illness constitutes a major problem [4], in particular due to the large numbers. Symptoms of stress can entail e.g. exhaustion, cognitive problems and poor sleep, which in turn reduce well-being and the capacity to manage activities in the everyday life of those affected [5].

The importance of combating mental health issues in people in working age has been emphasized [2]. Primary care interventions exist, e.g. behavioral and multimodal interventions [6] and occupational therapy interventions aimed at lifestyle changes, and skill-building to promote health [7]. Patients with stress-related disorders may also be offered interventions, e.g. group treatment programs to facilitate changes in everyday life [8], nature-based interventions to facilitate the reduction of symptoms of stress [9], or interventions focusing on work rehabilitation [10, 11].

The primary care services form the first line of care in Sweden, and according to Swedish Government, they should coordinate not only interventions for reducing symptoms, but also focus on interventions, which are health-promoting, preventive and rehabilitative, in order to increase the health of the population [12].

This study protocol presents a health-promoting intervention focusing on improving health and well-being as well as on activity-related aspects among people with a diagnosed stress-related disorder, by addressing their well-being and what contributes to this from their own perspective. The present intervention originates from a pilot project designed by the research group “Finding viability in daily life, experiences of well-being despite stress-related illness” [13, 14] using photo elicitation as a visual research method. Previous research has found visual research methods useful in relation to complex phenomenon [15, 16], such as well-being. Pictures present more and other information than words do [17] and allowing the participants to photograph by themselves has been described as to “capture the world through the participant’s eyes” (18, p.1).

The results in our previous pilot project [13, 14], with twelve women with stress-related illness, showed that experiences of taking photographs and talking about well-being in their everyday life, based on these photographs, was considered valuable as it redirected the participants towards a focus on their well-being [14]. Furthermore, it was found that well-being was perceived as an unconditional beingness, when the participants did not have to perform or meet any demands. A supportive environment was found to be important for promoting well-being, while some participants developed their own strategies to achieve the same aim [13].

The pilot project did not aim to be therapeutic, but positive feelings about participation and the usefulness of taking photographs and talking about them, were described. However, participants also described a need for continued training based on the new focus on well-being.

In summary, the results of the pilot project indicated that using photographs for reflecting about well-being in everyday life, despite suffering from stress-related disorders, was perceived as useful. The promising results inspired us to evaluate whether an intervention based on photo-supported conversations about well-being, conducted over time for increased training, could be beneficial as a health-promoting supplement to care as usual. Such a type of intervention, as a complement to stress rehabilitation programs, might be useful for health professionals working with people suffering from stress-related disorders or at risk for this.

However, the pilot project only consisted of one occasion for reflections about wellbeing based on photographs, and we have no knowledge about the potential outcomes of a health-promoting intervention consisting of several sessions. We thus want to investigate patients’ self-rated health and well-being, activities in everyday life, and potential improvements from a quantitative as well as qualitative perspective. This study protocol concerns a health-promoting intervention for the intervention group, named Be Well™, based on photo-supported conversations aiming to improve health and well-being, and focusing on quantitative as well as qualitative outcomes, compared with a relevant control group.

## Methods

### Aim

The aim of this study is to evaluate whether photo-supported conversations about well-being (Be Well™) as an intervention, in addition to care as usual within the primary care services, improves health and well-being for patients with stress-related illnesses. The intervention will be compared to a control group, who receive care as usual. A further aim is to conduct a process evaluation.

The research questions related to the quantitative evaluation are:

- How are the participants’ health and well-being affected by photo-supported conversations?
  o Primary outcome is self-rated symptoms of exhaustion.
  p Secondary outcomes are self-rated, depression and anxiety, sense of coherence and quality of life.
- How are the participants’ activities in everyday life affected by the photo-supported intervention?
  o Secondary outcomes are self-rated balance of activities in everyday life and work ability.
    The research questions related to the qualitative evaluation are:
- How are the participants’ well-being and everyday life experienced and affected by photo-supported conversations?
  o How do the participants perceive their well-being and everyday life, prior to and after the intervention?
  p How can the photographs contribute to the understanding of well-being in everyday life?
    The research questions related to the process evaluation of the intervention Be Well™ are:
- How do the participants perceive their participation in the photo-supported conversations about well-being?
- How do the participants rate their client satisfaction?
- How do the therapists perceive the performance of photo-supported conversations about well-being?

### Design and setting of the study

This project is a quasi-experimental pre-test post-test intervention with a control group [19] using quantitative as well as qualitative methods.

### Participants

The inclusion criteria are men and women between 20 and 67 years, diagnosed with the stress related ICD-10 diagnoses F43.8 and F43.9 [20], and with contact in primary care due to their symptoms of stress. Exclusion criteria are severe somatic disorders, neuropsychiatric diagnosis, psychosis and language or cognitive problems that implies difficulties in completing questionnaires.

### Context and procedure

The project takes place within the primary healthcare services in two regions in southern Sweden. Five primary care centres recruit participants to the intervention group while five others recruit participants to the control group. Primary care centres who have shown interest in the project have been engaged, and the units themselves chose whether to participate as part of the intervention group or the control group. The primary care centres, however, are comparable in terms of the size of cities where they are situated, socio-economic conditions, and provision of treatment methods. Both groups of participants receive their care as usual in the primary care context.

Potential participants who meet the criteria receive information about the project from a rehabilitation coordinator. When a patient declines to participate, the rehabilitation coordinator informs the project leader about their sex, age, diagnosis, and when possible the reason for declining. This is carried out with the aim of facilitating the exploration of potential differences between those who choose to participate and those who choose not to. When a patient is interested in participating, the rehabilitation coordinator forwards their contact information to the project leader and one of the researchers/project assistants contacts them for a meeting where they receive further information and can ask questions about the study and what participation entails. When agreeing to participate, the patients sign an informed consent and baseline data is collected.

#### Education prior to the intervention delivery

Five therapists, all of who are occupational therapists (OT), conduct the intervention Be-Well™, i.e. photo-supported conversations about well-being. Prior to commencing the intervention, they have participated in a course lasting two days, including discussions about the method and its background. There was a two-week interval between the first and second days and the education was performed by the research group (ABG, PW, HTS, UH). The OTs received information about the previous pilot project and its results during the course. The topics of health and well-being [21–24] well-being despite stress-related illness [13, 14], balance in activities in everyday life [25] and visual methodology [15] were also addressed and discussed. Moreover, the topic of photography and pictures was presented together with related ethical issues by a researcher (HTS) with knowledge in the field of visual studies, who also gave a lecture on photo interpretation. The training also involved practical exercises where the OTs tried the method of photographing well-being and talking about their own well-being with each other, based on their photographs, and acting both as a patient and as the therapist.

The OTs also took new pictures about their well-being between the first and the second day and had new conversations about these. Their experiences from these were then focused on in group discussions and reflected upon on several occasions. The intervention and its different sessions were described and discussed in detail so that all therapists had good knowledge of it at the end of the two-day course. The OTs discussed with each other and with the researchers throughout the course, and at the end of the second day, they had further opportunities to get answers to any remaining questions and solutions for any issues that were outstanding.

The project leader has continuous contact with the OTs to discuss their progress and clarify potential issues after the education and throughout the project. The OTs also meet regularly to discuss together with the researchers and the project assistants.

### The intervention

The intervention group received photo-supported conversations about well-being, i.e. the intervention Be-Well™, in addition to care as usual. The intervention has been developed on the basis of previous studies in the pilot project [13, 14], and on knowledge about health, well-being and everyday life activities [23, 24], and visual methodology [15].

The intervention is individually conducted, and the patients meet their occupational therapist (OT) physically and/or virtually for 12 sessions (intended to be conducted within 12 to 15 weeks). The intervention consists of three parts, sessions 1–4, sessions 5–10 and, sessions 11–12.

Sessions 1 to 4 are full-length 60-min meetings with the therapist. They were originally intended to be physical meetings but due to the Covid-19 pandemic they can also be switched to be virtual. The patient takes photographs of what they see as being related to their well-being prior to each of the sessions and sends 1–3 of these photographs to the OT before the session. The OT enlarges each of the photographs to the A4 size and brings them to the session. The conversation during the session is based on the photographs and the patient is encouraged to talk about each of them and how they relate to their well-being as well as the feelings or thoughts related to the photograph. Potential strategies for everyday life are discussed, e.g. to do more or less of an activity which relates to their well-being or solve a difficult activity in another way, and these strategies are tested between the sessions. Sessions 2 to 4 begin with a reflection on the last session before the new photographs are discussed.

Sessions 5–10 are short virtual meetings of approximately 15–20 min with the aim of following the patient, confirming their process and supporting them in their direction towards increased well-being. The patient continues photographing well-being, discusses these photographs shortly with their OT as well as their self-given homework to increase their well-being.

The final two sessions are 60-min physical or virtual meetings where the aim is to compile the patient’s experiences of well-being together with the previous sessions and to formulate their continued activity. The patient is encouraged to “look in the rear-view mirror” during session 11 by viewing and reflecting upon all their previous photographs, which are placed in front of them. The intention is to look forward by reflecting upon their photographs during session 12. The patient is asked what he/she wants to take away from the intervention as well as being recommended to give themselves advice about how to keep or find situations in their everyday life that are of importance for their well-being. The patient is also asked to choose one or two of their enlarged pictures to use as a reminder of everyday life or as a future goal.

### Control

The control group receives their care as usual, e.g. medication, physiotherapy, psychotherapy, occupational therapy and/or vocational interventions. The therapists who recruit patients (nurses, OTs, psychologists and social workers), also meet regularly to discuss the recruiting with the researchers and project assistants.

### Data collection

Two researchers (ABG, UH), and one project assistant collect the data for the intervention group and three project assistants collect data for the control group. The project assistants are OTs, who are not involved in the intervention. All project assistants have been informed about the project and its aim and the project leader will have continuous contact with them throughout the project to discuss progress and clarify potential issues. The project assistants also meet regularly to discuss together with the researchers and the therapists.

**Fig. 1:**
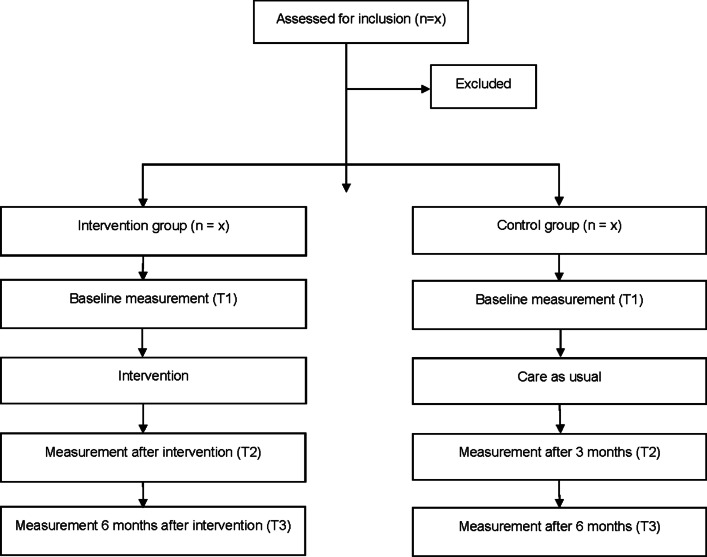
Overview of the procedure

The intervention group meets the data collector prior to the start of the intervention; after the intervention; and 6 months after the performed intervention ended. The control group meets the data collector at the same time points as the intervention group (Fig. 1). In order to protect their confidentiality, all the participants received a code number when recruited, and the list linking names to code numbers is stored separately.

#### Data material

The data in this project consists of questionnaires, interviews and photographs.

#### Quantitative measures

Participants in the intervention and in the control group answer the same questions including gender, age, diagnosis, social network, employment, sick leave/not sick leave, work situation and degree of paid work, physical activities, and care consumption. As the project takes place during the pandemic Covid-19, the participants are also asked about whether and how they are affected by the pandemic in their personal lives, as well as in relation to their paid work.

##### Primary outcomes

*Health related aspects* are measured in terms of; *Symptoms of exhaustion,* using the Karolinska Exhaustion Disorder Scale (KEDS) [26]. The instrument has nine items measuring symptoms of exhaustion, cognitive problems, sleeping, and tolerance to further stress on a seven-step ordinal scale from 0 to 6 where higher is worse and is summed with a cut-off of 19. The instrument originates from Sweden and has shown good psychometric properties [26].

##### Secondary outcomes

*Health and well-being related aspects* are measured in terms of; *Symptoms of depression and anxiety, quality of life, sense of coherence, and client satisfaction. Depression and anxiety* is measured with the Hospital Anxiety and Depression Scale (HADS) [27]. It consists of seven items measuring symptoms of depression and anxiety respectively, and can be measured as a whole, or forming two sub-scales. Each item is measured on a four-step ordinal scale from 0 to 3 where higher is worse. The Swedish version of the instrument is commonly used and found appropriate [28, 29].

*Quality of life* is measured by using the Manchester Short Assessment of quality of life (MANSA) [30]. The instrument is interview-based and has twelve items that measure satisfaction with life, and aspects of life as accommodation, work, leisure, relationships, accommodation, and physical and mental health on a seven step ordinal scale from 1 to 7 where higher is better and can be summed. The Swedish version of the instrument has previously shown good psychometric properties [31].

*Sense of coherence* is measured with the Sense of Coherence Scale (SoC) [32]. The 13 items scale measures how well individuals are coping with life stress and psychological stress on seven-steps ordinal scales from 1 to 7, higher is better and it can be summed. The Swedish version of the instrument is frequently used and has shown good psychometric properties [33].

*Activity related aspects* are measured in terms of: balance of activities in everyday life and work ability. *Balance of activities in everyday life* is measured by using the Occupational Balance Questionnaire (OBQ11) [34], which focuses on satisfaction with the amount and variation of occupations in everyday life. The OBQ11consists of eleven items measured on a four-step ordinal scale from 0 to 3 where higher is better and can be analysed on item level and as a summed scale. The instrument originates from Sweden and has shown good psychometric properties [34, 35].

*Work ability* is measured with the Worker Role Self-assessment (WRS-18) [36]. This instrument measures self-rated perceptions of current and future work and work role. The instrument has 18 items measured on five-step ordinal scales from 1 to 5 where higher is better. The instrument originates from Sweden and has shown good psychometric properties [37].

*Client satisfaction* is measured with the Client Satisfaction Questionnaire (CSQ) [38]. The instrument has eight items and measures satisfaction with received treatment on a four-step ordinal scale from 1 to 4 where higher is better. It has previously been found to have good psychometric properties [39, 40] in a Swedish context [41].

#### Qualitative measures

Participants in the intervention group are interviewed individually on all data collections with open-ended questions [42] focused on their experiences of well-being in their present life situation and follow-up questions to gain a greater understanding of their life situation. Participants in the control group are interviewed individually during a shorter interview, focusing on their present life situation. Both groups are interviewed three times.

The interview *prior to the intervention* is related to the participant’s present situation in everyday life, and what causes stress at home and at work, and what contributes to well-being despite living with stress.

The interview *after the intervention* in addition to the same open-ended questions as prior to the intervention, also focuses on the participant’s experiences of the intervention to increase health, well-being and work ability. During this occasion, their photographs from the intervention are used as well and copies of the photographs are also handed over to the data collector as they are data.

The interview *six months after the intervention* focuses on the participant’s present life situation, as well as their perception of photo-supported conversations as intervention. The interview also focused on whether, and if so, how they have been able to use photographs in their everyday life to achieve well-being after the intervention ended.

Each interview is audio recorded and begins with open questions and the participant is encouraged to talk about how they achieve well-being in relation to their everyday life, by describing a certain situation or example. Follow up questions are used as well.

All therapists will, after the final participant, who is receiving their intervention has finished, also be asked to participate in an interview (individual and/or focus group) for investigating their experiences of the intervention, and how to improve the intervention in order to better suit clients with stress-related disorders.

### Sample size

The power calculation for this study was based on the Karolinska Exhaustion Disorder Scale (KEDS) [26]. In a previous study where this instrument was used, the response within each subject group was normally distributed with standard deviation 8.74 [43]. It was thus anticipated that if the true difference in KEDS means (scale on 0–54) between the experimental and control is 7, we will need to study 29 experimental subjects and 29 control subjects to be able to reject the null hypothesis that the population means of the experimental and control groups are equal with probability (power) 0.8. As this study has several timepoints, it is likely that there will be dropouts from the study, and we will thus include a total of 35 experimental subjects and 35 control subjects. The Type I error probability associated with this test of this null hypothesis is 0.05.

### Analysis

Non-parametric statistics will be used for investigating potential differences between the intervention and control groups in health, and activity-related variables as well as in client satisfaction since the instruments are based on nominal and ordinal scales. Missing data for dropouts will be estimated using the last data obtained before a participant dropped out. The transcribed interviews will be analysed for meanings using thematic [44] and phenomenological [42] analysis. The qualitative data will be analysed for meanings in accordance with the methodological principles of openness, flexibility and a reflective attitude. The analysis is a process defined by a movement between the whole and the parts, with the purpose of identifying a new whole in the phenomenological studies [42] and identifying themes of meanings in the thematic studies [44]. The photographs will be analysed with a semiotic method [45] regarding content and formal aspects, such as shape and color, perspective, spatiality and composition.

### Time plan

The inclusion process began in 2020 and is planned to be completed in 2022. The intervention started in proximity to the inclusion and all data will be collected about six months after the intervention ended for the last participant.

## Discussion

Stress-related illnesses are common and negatively affect both the individuals and society [3–5]. Experiencing less exhaustion and a satisfying everyday life is important for this group as well as continuing or returning to work [7, 46, 47], but more research is needed concerning health-promoting interventions. The present project is based on the concept [13, 14] that focusing on well-being despite living with a stress-related illness may positively impact health and everyday life aspects, and that the photo-supported conversations about well-being, Be Well™, can contribute a valuable complement to other treatment and rehabilitation strategies. A strength is the use of varying methodologies, such as quantitative measures, photographs, and qualitative interviews with participants and therapists providing broad complementary information about what happens longitudinally. The results of the project will thus provide some first insights into the potential effect of this method. However, it should be noted that all the therapists in the present study were OTs but the intervention is not considered to be solely an OT intervention. Further research with other professions as therapists is needed.

There are some potential limitations and difficulties that may be experienced in carrying out this study. For people living with stress-related disorders, participating in a research study might involve a risk of not acknowledging when they need to have a break and rest. To minimize this risk voluntariness, and the opportunity to withdraw at any time, will be highlighted. The benefit of participating in the study, based on its focus on well-being, was seen to counteract the potential risks. Further, participating in a research study can entail extra strain, which can mean that we cannot recruit those who are too stressed. Furthermore, it may be difficult to control for other treatments. In order to gain knowledge about the content of care as usual, all therapists, in the experimental group as well as in the control group, before entering the research study, reported the content of care as usual at their primary healthcare service. Furthermore, to ensure that the therapists follow the guidelines, the research group offers regularly meetings to discuss the performance of the research. Moreover, a standardised protocol has been developed for assessing the adherence to photo-supported conversations. The therapists who carry out the intervention report the content of each session after each completed intervention.

## Data Availability

The datasets generated and analysed during the present study are not publicly available due to ethical considerations but could be available from the corresponding author on request.

## Abbreviations

Be Well™: The intervention based on photo supported conversations
CSQ: Client satisfaction questionnaire
HADS: Hospital anxiety and depression scale
KEDS: Karolinska Exhaustion Disorder Scale
MANSA: Manchester short assessment of quality of life
OBQ11: Occupational balance questionnaire
SOC: Sense of coherence
WRS-18: Worker Role Self-assessment
